# Ethnic differences in risk of severe Covid-19: To what extent are they driven by exposure?

**DOI:** 10.1093/pubmed/fdab347

**Published:** 2021-09-21

**Authors:** Rhiannon Edge, Diana A van der Plaat, Vaughan Parsons, David Coggon, Martie van Tongeren, Rupert Muiry, Paul Cullinan, Ira Madan

**Affiliations:** Lancaster Medical School, Lancaster University, Bailrigg, Lancaster LA1 4YW, UK; National Heart and Lung Institute (NHLI), Imperial College London, London SW3 6LY, UK; Occupational Health Service, Guy’s and St Thomas NHS Foundation Trust, London SE1 7EH, UK; Faculty of Life Sciences and Medicine, King’s College London, London SE5 9RJ, UK; MRC Lifecourse Epidemiology Centre, University of Southampton, Southampton SO16 6YD, UK; Centre for Occupational and Environmental Health, School of Health Sciences, University of Manchester, Manchester M13 9NT, UK; Occupational Health Service, Guy’s and St Thomas NHS Foundation Trust, London SE1 7EH, UK; National Heart and Lung Institute (NHLI), Imperial College London, London SW3 6LY, UK; Occupational Health Service, Guy’s and St Thomas NHS Foundation Trust, London SE1 7EH, UK; Faculty of Life Sciences and Medicine, King’s College London, London SE5 9RJ, UK

**Keywords:** Covid-19, ethnicity, risk, sickness absence, vulnerability

## Abstract

**Background:**

This study quantifies the risk of Covid-19 among ethnic groups of healthcare staff during the first pandemic wave in England.

**Methods:**

We analysed data on 959 356 employees employed by 191 National Health Service trusts during 1 January 2019 to 31 July 2020, comparing rates of Covid-19 sickness absence in different ethnic groups.

**Results:**

In comparison with White ethnic groups, the risk of short-duration Covid-19 sickness absence was modestly elevated in South Asian but not Black groups. However, all Black and ethnic minority groups were at higher risk of prolonged Covid-19 sickness absence. Odds ratios (ORs) relative to White ethnicity were more than doubled in South Asian groups (Indian OR 2.49, 95% confidence interval (CI) 2.36–2.63; Pakistani OR 2.38, 2.15–2.64; Bangladeshi OR 2.38, 1.98–2.86), while that for Black African ethnicity was 1.82 (1.71–1.93). In nursing/midwifery staff, the association of ethnicity with prolonged Covid-19 sickness absence was strong; the odds of South Asian nurses/midwives having a prolonged episode of Covid-19 sickness absence were increased 3-fold (OR 3.05, 2.82–3.30).

**Conclusions:**

Residual differences in risk of short term Covid-19 sickness absences among ethnic groups may reflect differences in non-occupational exposure to SARS-CoV-2. Our results indicate ethnic differences in vulnerability to Covid-19, which may be only partly explained by medical comorbidities.

## Introduction

The disproportionate impact of the Covid-19 pandemic on minority ethnic groups in the UK is now well established^1^ but not fully understood. During the first wave (24 January 2020 to 11 September 2020), people from all ethnic minority groups (except for women in the Chinese or ‘White Other’ ethnic groups) had higher rates of death involving SARS-CoV-2 than the White British population. The rate was highest for the Black African group (3.7 times greater than for the White British group for males, and 2.6 greater for females), followed by the Bangladeshi (3.0 for males, 1.9 for females), Black Caribbean (2.7 for males, 1.8 for females) and Pakistani (2.2 for males, 2.0 for females) ethnic groups.

**Figure:**
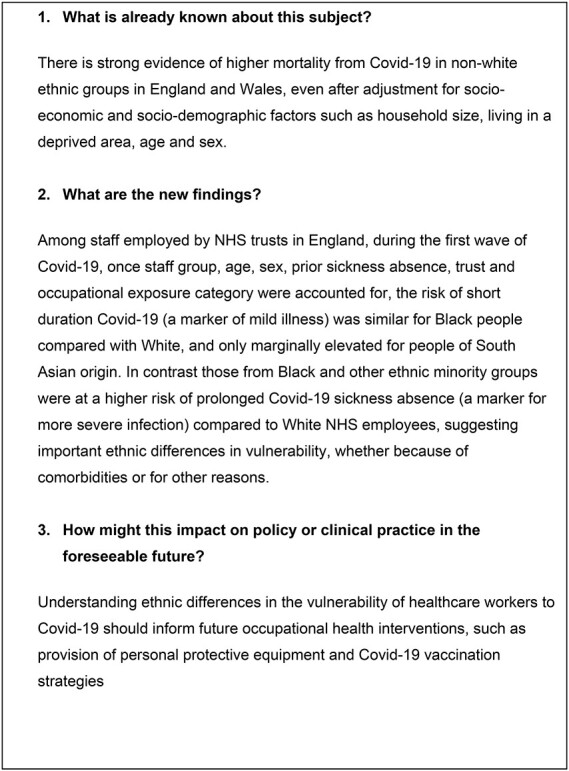


These findings could arise from differences in exposure to infection and/or differences in vulnerability to more severe disease when infection occurs. Vulnerability to Covid-19 is related to age, sex and various comorbidities. One factor that contributes to exposure to SARS-CoV-2 infection is occupation. If minority ethnic groups were employed disproportionately in occupations entailing proximity to other people, particularly people who are more likely to be infected with SARS-CoV-2, then they would be at higher risk of infection. Exposure to infection will depend also on other factors such as household size and composition, housing density, and non-occupational activities and behaviours.^2^ Large record linkage studies such as OpenSAFELY suggest that important differences in mortality by ethnicity persist even after allowance for region, social deprivation, sex, age and multiple comorbidities.^3^ However, it remains possible that there are differences in exposures through work, and to date, few studies have been able to adjust well for occupational differences in exposure.

The aim of our study was to determine whether ethnic differences in risk of less serious Covid-19 (which is less likely to be influenced by differences in vulnerability) were apparent during the first wave of the pandemic among healthcare workers in England in specific job categories, after adjustment for potential exposure to infected patients and geographical variation in rates of infection.

## Methods

As detailed in an earlier report,^4^ we analysed pseudonymized data abstracted from the National Health Service (NHS) electronic staff record (ESR) for all personnel who had been continuously employed by NHS trusts in England during 01 January 2019 to 31 July 2020.

In the analysis for this paper, we focused on two main outcomes—(i) Covid-19 sickness absence beginning between 09 March 2020 and 16 July 2020, at least one episode of which was prolonged (i.e. with duration >14 days); and (ii) Covid-19 sickness absence during the same period that was only ever of shorter duration. Covid-19 sickness absence was defined as sickness absence ascribed to any of five diagnostic categories (cough/flu, chest/respiratory, infectious diseases, other and unknown) with Covid-19 recorded as a related reason.

The main explanatory variables of interest were ethnicity and staff group. Ethnicity was classified initially to the 12 categories listed in Table 1, but in some analyses, we aggregated all South Asian ethnic groups and all Black ethnic groups to ensure statistically meaningful numbers. Staff group was classed to nine categories (Table 1), following a scheme that was employed in the ESR, but with students aggregated into a category labelled as ‘Other or unknown’, which also included some individuals who held multiple jobs simultaneously. As in our earlier report,^4^ where individuals had changed staff group over the study period, we aimed to classify them according to the job held on 09 March 2020.

**Table 1:** Numbers of subjects in staff group categories at 9 March 2020 according to ethnicity

*Ethnic group*	*Staff group*									
	*Administrative and clerical*	*Additional clinical services*	*Additional professional scientific and technical*	*Allied health professionals*	*Estates and ancillary*	*Healthcare scientists*	*Medical and dental*	*Nursing and midwifery registered*	*Other or unknown (including multiple)*	*All categories*
White	172 338	146 525	33 951	63 646	48 072	16 802	42 124	204 859	3091	731 408
	23.6%	20.0%	4.6%	8.7%	6.6%	2.3%	5.8%	28.0%	0.4%	100.0%
Indian	6842	5611	2401	2037	2211	1234	13971	13 458	100	47 865
	14.3%	11.7%	5.0%	4.3%	4.6%	2.6%	29.2%	28.1%	0.2%	100.0%
Pakistani	2673	2165	858	778	452	662	4406	1666	47	13 707
	19.5%	15.8%	6.3%	5.7%	3.3%	4.8%	32.1%	12.2%	0.3%	100.0%
Bangladeshi	1721	924	288	171	137	177	677	546	20	4661
	36.9%	19.8%	6.2%	3.7%	2.9%	3.8%	14.5%	11.7%	0.4%	100.0%
Other or unspecified South Asian	102	97	25	23	37	30	267	67	0	648
	15.7%	15.0%	3.9%	3.5%	5.7%	4.6%	41.2%	10.3%	0.0%	100.0%
Other or unspecified Asian	2646	7393	1318	1052	2114	863	5047	19 089	63	39 585
	6.7%	18.7%	3.3%	2.7%	5.3%	2.2%	12.7%	48.2%	0.2%	100.0%
Black—African	3757	8268	1110	1160	2153	732	2685	16 383	184	36 432
	10.3%	22.7%	3.0%	3.2%	5.9%	2.0%	7.4%	45.0%	0.5%	100.0%
Black – Caribbean	4316	3797	486	449	1027	168	248	4349	66	14 906
	29.0%	25.5%	3.3%	3.0%	6.9%	1.1%	1.7%	29.2%	0.4%	100.0%
Black—other or unspecified	1177	1166	158	157	362	99	267	1748	22	5156
	22.8%	22.6%	3.1%	3.0%	7.0%	1.9%	5.2%	33.9%	0.4%	100.0%
Mixed	3341	3435	876	1162	960	379	2357	4413	96	17 019
	19.6%	20.2%	5.1%	6.8%	5.6%	2.2%	13.8%	25.9%	0.6%	100.0%
Other	1217	2429	523	420	798	285	2945	4785	32	13 434
	9.1%	18.1%	3.9%	3.1%	5.9%	2.1%	21.9%	35.6%	0.2%	100.0%
Unknown	6256	6479	1231	1846	3512	776	5276	9058	101	34 535
	18.1%	18.8%	3.6%	5.3%	10.2%	2.2%	15.3%	26.2%	0.3%	100.0%
All ethnic groups	206 386	188 289	43 225	72 901	61 835	22 207	80 270	280 421	3822	959 356
	21.5%	19.6%	4.5%	7.6%	6.4%	2.3%	8.4%	29.2%	0.4%	100.0%

In addition, we considered five other explanatory variables—trust (191 categories) sex, age group (8 categories), number of episodes of sickness absence in 2019 (4 categories) and exposure category. The last was assigned by application of a job-exposure matrix to the occupation (659 possible categories) that the individual held on 09 March 2020. It was assigned to two levels according to whether or not the occupation was judged to involve face-to-face or hands-on care of patients who were more likely to have Covid-19 than the general population. In earlier analyses, such exposure was associated with clearly elevated risk of Covid-19 sickness absence.^4^ The other variables were classified as in our previous report.^4^

Statistical analysis was carried out using R statistical software. We used logistic regression to estimate odds ratios (ORs) with 95% confidence intervals (CIs) for the two outcomes in relation to combinations of ethnicity and staff group with adjustment for other explanatory variables.

Ethical approval to conduct the study was obtained from the NHS Health Research Authority (reference 20/SC/0282).

## Results

After exclusion of 3811 employees who were absent from work continuously between 09 March 2020 and 31 July 2020 (mainly because of maternity or study leave), analysis was based on 959 356 individuals (77% female) from 191 trusts. Most (89%) were aged between 25 and 60 years. Detailed information on the numbers of individuals by age band and by frequency of sickness absence during 2019 has been reported elsewhere.^5^ From application of the job-exposure matrix, 383 097 (39.9%) employees held jobs at 09 March 2020, which were classed as providing hands-on or face-to face care for patients who could be expected to have a higher prevalence of Covid-19 than the general population. Table 1 shows the distribution of the study sample according to staff group at 9 March 2020 and ethnic group. Among staff of Asian ethnicity, the proportion employed as doctors or dentists was some five times higher than in White workers. Relatively high proportions of the Black ethnic groups, and especially Black African, were registered nurses or midwives.

In total, 20 988 individuals (2.2%) had at least one episode of Covid-19 sickness absence that started between 09 March 2020 and 16 July 2020 and continued for >14 days (prolonged Covid-19 sickness absence). In addition, a further 70 863 (7.4%) had episodes of Covid-19 sickness absence during that period, all of which were of shorter duration.

Table 2 shows associations of Covid-19 sickness absence with ethnicity and staff group, according to whether absence was only ever of short duration (≤14 days), or at least one episode was prolonged. In comparison with White ethnicity, the risk of short-duration Covid-19 sickness absence was modestly elevated in Indian (OR 1.23 95% CI 1.18–1.27), Pakistani (OR 1.10 95% CI 1.03–1.17), Bangladeshi (OR 1.17 95% CI 1.04–1.31) and Asian (OR 1.41 95% CI 1.36–1.46) ethnic groups. However, all Black and ethnic minority groups were at higher risk of prolonged Covid-19 sickness absence, and to a greater extent. In particular, ORs relative to White ethnicity were more than doubled for those in the South Asian ethnic groups (Indian OR 2.49, 95% CI 2.36–2.63; Pakistani OR 2.38, 95% CI 2.15–2.64; Bangladeshi OR 2.38, 95% CI 1.98–2.86), while that for Black African ethnicity was 1.82 (95% CI 1.71–1.93).

**Table 2:** Associations of ethnicity and staff group with Covid-19 sickness absence according to maximum duration of episodes

*Risk factor*	*Covid-19 sickness absence during study period*						
	*None*	*All episodes ≤14 days*			*At least one episode >14 days*		
	*N*	*N*	*OR*	*(95% CI)*	*N*	*OR*	*(95% CI)*
Ethnicity							
White	668 583	50 330	*ref.*	*ref.*	12 495	*ref.*	*ref.*
Indian	41 961	4093	1.23	1.18–1.27	1811	2.49	2.36–2.63
Pakistani	12 192	1090	1.10	1.03–1.17	425	2.38	2.15–2.64
Bangladeshi	4188	348	1.17	1.04–1.31	125	2.38	1.98–2.86
South Asian—not further specified	583	50	1.01	0.75–1.37	15	1.53	0.91–2.59
Asian—other or unspecified	32 227	5085	1.41	1.36–1.46	2273	2.69	2.55–2.83
Black—African	31 866	3144	1.04	1.00–1.08	1422	1.82	1.71–1.93
Black—Caribbean	13 398	1057	0.91	0.85–0.97	451	1.38	1.25–1.52
Black—other or unspecified	4565	410	1.00	0.90–1.11	181	1.65	1.42–1.93
Mixed	15 192	1442	1.08	1.02–1.15	385	1.37	1.23–1.52
Other	11 466	1346	1.24	1.17–1.32	622	2.28	2.09–2.49
Unknown	31 284	2468	1.01	0.97–1.06	783	1.27	1.18–1.37
Staff group at 9 March 2020							
Administrative and clerical	195 265	8781	*ref.*	*ref.*	2340	*ref.*	*ref.*
Additional clinical services	164 592	17 549	1.82	1.77–1.88	6148	2.14	2.03–2.26
Additional professional scientific and technical	40 309	2407	1.38	1.32–1.45	509	1.15	1.04–1.27
Allied health professionals	65 421	6288	1.66	1.59–1.72	1192	1.27	1.18–1.37
Estates and ancillary	57 201	3422	1.40	1.34–1.46	1212	1.60	1.49–1.72
Healthcare scientists	20 737	1229	1.19	1.11–1.26	241	0.92	0.8–1.05
Medical and dental	74 134	5075	1.43	1.37–1.48	1061	0.85	0.78–0.92
Nursing and midwifery registered	246 380	25 809	1.81	1.76–1.86	8232	1.84	1.75–1.95
Other or unknown (including multiple)	3466	303	1.49	1.32–1.69	53	1.33	1.00–1.75

Risk estimates are relative to no Covid-19 sickness absence during study period and were derived from two logistic regression models (one per outcome), each of which also included trust (200 categories), sex, age group (8 categories), number of episodes of sickness absence in 2019 (4 categories) and exposure category at 9 March 2020 (two categories)—for further detail, see text.

*ref.* is in italics in the table, this denotes the reference group for the odds ratios, but again this does not need a footnote.

Table 3 presents risk estimates by ethnic group for Covid-19 sickness absence that was only ever of short duration, when analyses were restricted to specific staff groups. To ensure adequate numbers, for this analysis we aggregated all South Asian ethnic groups and all Black ethnic groups. The reference was no Covid-19 sickness absence at any time during the study period. The higher risks of short-duration Covid-19 sickness absence in Asian and/or South Asian ethnic groups were apparent in most staff groups but were not observed among doctors and dentists (OR 0.99, 95% CI 0.92–1.07).

**Table 3:** Associations of ethnicity with short-duration Covid-19 sickness absence according to staff group

*Ethnic group*	*Staff group*							
	*Administrative and clerical*	*Additional clinical services*	*Additional professional scientific and technical*	*Allied health professionals*	*Estates and ancillary*	*Healthcare scientists*	*Medical and dental*	*Nursing and midwifery registered*
	*OR*	*OR*	*OR*	*OR*	*OR*	*OR*	*OR*	*OR*
	*(95% CI)*	*(95% CI)*	*(95% CI)*	*(95% CI)*	*(95% CI)*	*(95% CI)*	*(95% CI)*	*(95% CI)*
White	*ref.*	*ref.*	*ref.*	*ref.*	*ref.*	*ref.*	*ref.*	*ref.*
South Asian	1.16	1.30	1.08	0.93	1.24	1.12	0.99	1.38
	(1.05–1.27)	(1.21–1.4)	(0.92–1.26)	(0.81–1.06)	(1.04–1.48)	(0.92–1.37)	(0.92–1.07)	(1.31–1.46)
Asian—other or unspecified	1.26	1.53	1.65	1.33	1.65	1.07	0.98	1.45
	(1.07–1.48)	(1.42–1.65)	(1.35–2.02)	(1.09–1.62)	(1.40–1.95)	(0.81–1.41)	(0.87–1.10)	(1.38–1.52)
Black	1.04	0.91	1.22	1.03	0.79	1.14	0.90	1.04
	(0.94–1.15)	(0.85–0.98)	(0.99–1.49)	(0.86–1.22)	(0.66–0.93)	(0.89–1.47)	(0.77–1.06)	(0.99–1.10)
Mixed	1.08	1.08	1.25	1.21	1.08	1.18	1.00	1.06
	(0.92–1.26)	(0.96–1.21)	(0.94–1.67)	(0.99–1.47)	(0.83–1.39)	(0.78–1.78)	(0.85–1.19)	(0.95–1.17)
Other	1.22	1.34	1.11	1.10	0.75	1.25	1.06	1.29
	(0.96–1.56)	(1.18–1.53)	(0.79–1.57)	(0.79–1.53)	(0.54–1.03)	(0.79–1.97)	(0.90–1.23)	(1.17–1.41)
Unknown	1.00	1.04	0.93	0.93	0.86	1.40	0.99	1.04
	(0.88–1.14)	(0.95–1.14)	(0.72–1.22)	(0.78–1.11)	(0.71–1.03)	(1.04–1.87)	(0.87–1.13)	(0.96–1.12)

Risk estimates are for Covid-19 sickness absence that was only ever of short duration (≤14 days) relative to no Covid-19 sickness absence and are derived from separate logistic regression models for each staff group, which also included trust (200 categories), sex, age group (8 categories), number of episodes of sickness absence in 2019 (4 categories) and exposure category at 9 March 2020 (two categories)—for further detail, see text.

*ref.* is in italics in the table, this denotes the reference group for the odds ratios, but again this does not need a footnote.

Table 4 gives findings from analyses analogous to those for Table 3, but with at least one prolonged episode of Covid-19 sickness absence as the outcome. Within each staff group, risk was highest in the South Asian and/or the other/unspecified Asian ethnic groups, with ORs (relative to White) substantially higher than for short-duration Covid-19 sickness absence. In contrast to the findings for shorter duration Covid-19 sickness absence, Black people were at an increased risk (relative to White) of prolonged Covid-19 sickness absence in several staff groups.

**Table 4:** Associations of ethnicity with prolonged Covid-19 sickness absence according to staff group

*Ethnic group*	*Staff group*							
	*Administrative and clerical*	*Additional clinical services*	*Additional professional scientific and technical*	*Allied health professionals*	*Estates and ancillary*	*Healthcare scientists*	*Medical and dental*	*Nursing and midwifery registered*
	*OR*	*OR*	*OR*	*OR*	*OR*	*OR*	*OR*	*OR*
	*(95% CI)*	*(95% CI)*	*(95% CI)*	*(95% CI)*	*(95% CI)*	*(95% CI)*	*(95% CI)*	*(95% CI)*
White	*ref.*	*ref.*	*ref.*	*ref.*	*ref.*	*ref.*	*ref.*	*ref.*
South Asian	1.91	2.51	2.04	1.70	2.14	3.09	1.60	3.05
	(1.63–2.24)	(2.26–2.78)	(1.53–2.72)	(1.31–2.2)	(1.67–2.73)	(2.17–4.41)	(1.38–1.85)	(2.82–3.30)
Asian—other or unspecified	2.04	2.84	2.00	2.55	2.80	1.69	1.18	2.94
	(1.56–2.68)	(2.57–3.14)	(1.31–3.07)	(1.80–3.62)	(2.22–3.53)	(0.97–2.94)	(0.91–1.54)	(2.73–3.16)
Black	1.60	1.38	1.43	1.66	1.41	1.43	0.97	2.02
	(1.35–1.89)	(1.24–1.54)	(0.95–2.14)	(1.19–2.31)	(1.11–1.81)	(0.83–2.47)	(0.69–1.37)	(1.86–2.18)
Mixed	1.39	1.20	1.28	1.13	0.99	0.29	1.26	1.62
	(1.04–1.86)	(0.98–1.47)	(0.69–2.36)	(0.70–1.83)	(0.61–1.63)	(0.04–2.07)	(0.87–1.80)	(1.37–1.92)
Other	1.85	2.56	1.55	1.29	0.72	2.97	1.51	2.62
	(1.24–2.76)	(2.18–3.02)	(0.81–2.99)	(0.66–2.55)	(0.40–1.3)	(1.39–6.37)	(1.13–2.03)	(2.31–2.97)
Unknown	1.05	1.26	1.44	0.88	1.33	1.17	1.40	1.35
	(0.82–1.33)	(1.1–1.45)	(0.9–2.32)	(0.61–1.26)	(1.02–1.73)	(0.56–2.45)	(1.07–1.81)	(1.18–1.54)

Risk estimates are for at least one episode of Covid-19 sickness absence with duration > 14 days relative to no Covid-19 sickness absence and are derived from separate logistic regression models for each staff group, which also included trust (200 categories), sex, age group (8 categories), number of episodes of sickness absence in 2019 (4 categories) and exposure category at 9 March 2020 (two categories)—for further detail, see text.

*ref.* is in italics in the table, this denotes the reference group for the odds ratios, but again this does not need a footnote.

In sensitivity analyses, we repeated the calculations for Tables 2–4, after exclusion of 6854 individuals for whom one or more of age, sex or ethnicity was imputed because of inconsistencies in the raw data. The results, which are presented in Supplementary Tables S1–S3, were virtually unchanged.

## Discussion

### Main finding of this study

Our analysis confirms that during the first wave of Covid-19 in England there were differences between ethnic groups in risk of short and longer duration Covid-19 sickness absence among NHS staff. Once staff group, age, sex, prior sickness absence, trust and occupational exposure category were accounted for, the risk of short duration Covid-19 was similar for Black people compared with White and only marginally elevated for people of South Asian origin. In contrast, staff from Black and other ethnic minority groups were at a higher risk of prolonged Covid-19 sickness absence compared to White NHS employees, suggesting important ethnic differences in vulnerability, whether because of comorbidities or for other reasons.

### What is already known on this topic

Multiple population-based studies have suggested that people from both Black and South Asian ethnic groups face an increased risk of SARS-CoV-2 infection compared to White people.^6^^,^^7^ However, this increase in risk can be at least partially explained by differences in socio-economic circumstances such as household size, number of dependent children and living in a deprived area.^6^

A cohort study found that critical care admissions in the UK were more common in South Asian (OR 1.28, 95% CI 1.09–1.52), Black (OR 1.36, 95% CI 1.14–1.62) and other minority ethnic groups (OR 1.29, 95% CI 1.13–1.47) than White people.^8^ A study of UK Biobank participants found that Black and Asian participants were at an increased risk of Covid-19 hospitalization compared to White participants; adjusting for socioeconomic factors and cardiorespiratory comorbidities led to some attenuation, but not complete elimination, of the increased risk in Black (OR 2.38 95% CI 1.52–3.74) and Asian participants (OR 1.75 95% CI 1.08–2.85).^9^ However, unlike the work presented here, these studies did not adjust for occupational exposure.

### What this study adds

This large study is the first to examine the associations of ethnicity with Covid-19 sickness absence in UK healthcare workers while accounting for occupational group and potential for exposure to infected patients. The sample size of almost a million individuals gave the investigation high statistical power and allowed us to investigate ethnic groups in detail (e.g. separating workers of Indian and Pakistani origin). Occupational groups were analysed separately, and an attempt was made to adjust for occupational exposure by using a bespoke job-exposure matrix. The effect of geographical differences in exposure to infection was accounted for by adjustment for hospital trust.

We explored the risk of short-duration sickness absence attributed to Covid-19 among NHS staff as a proxy for less serious Covid-19, which is less likely to be influenced by differences in vulnerability. By adjusting for the potential occupational exposure to infected patients (assessed by the job-exposure matrix), as well as trust (a specific geographical marker), sex and age, we have shown that any differences in risk of mild Covid-19 by ethnicity were small. The residual variation may reflect differences in exposure that were not adequately captured by staff group and exposure category.

In contrast, the difference in risk of prolonged Covid-19 among Black and ethnic minority groups compared to White was more exaggerated than for short-duration Covid-19 sickness absence. Within each staff group, the risk of prolonged Covid-19 sickness absence was highest in the South Asian and/or the other/unspecified Asian ethnic groups, and often the odds were twice those of White people. Our findings that ethnic minority groups are at higher risk of severe Covid-19 are supported by several other studies.

In our study, ethnic disparities in short-duration Covid-19 sickness absence were not observed among those employed as healthcare scientists or doctors and dentists, in contrast to those employed in other roles within the NHS. It may be that non-occupational risk factors for infection differ less by ethnicity within these groups than in other job groups. Within healthcare scientists, doctors and dentists, ethnic differences were apparent, however, for longer duration Covid-19 sickness absence, again suggesting differences in vulnerability to severe illness when infection occurs.

### Limitations of this study

Ethnicity was coded in the ESR with varying degrees of specificity and not always consistently. Exposure category was defined based on employment at 9 March 2020 and did not capture redeployment to different clinical settings during the pandemic. We were not able to account for use of personal protective equipment which may have biased our analysis if it differed by ethnicity within job groups. A British Medical Association snapshot survey taken early in the first wave of the pandemic suggested that a higher proportion (68%) of doctors from minority ethnic groups felt pressured to work with inadequate personal protective equipment where aerosol-generating procedures were being carried out, than those who identified as White (33%).^10^ A further limitation is that sickness absence is an imperfect marker for the occurrence of Covid-19, and it is possible both that true cases were missed (due to asymptomatic illness) and that other respiratory illnesses were sometimes incorrectly attributed to coronavirus. However, our previous analysis showed that Covid-19 sickness absence correlated with seropositivity for SARS-Cov-2.^4^

## Supplementary Material

Supplementary_Tables_S1-3_fdab347

Strobe_Checklist_Paper_2_1_0_fdab347

## Data Availability

With permission, source data are available upon request from the NHS Electronic Staff Record (ESR) Warehouse (NHS England).

## Notes

**Rhiannon Edge,** Lecturer

**Diana A. van der Plaat,** Research Fellow

**Vaughan Parsons,** Research Manager

**David Coggon,** Professor

**Martie van Tongeren,** Professor

**Rupert Muiry,** Research Assistant

**Ira Madan,** Professor

**Paul Cullinan,** Professor
