# Supplementary material for: Ethnic differences in risk of severe Covid-19: To what extent are they driven by exposure?
Source: J Public Health (Oxf). 2021 Sep 21;44(4):787–96. doi: 10.1093/pubmed/fdab347 (PMC8500021; doi:10.1093/pubmed/fdab347)
Supplement: Supplementary_Tables_S1-3_fdab347 [file supplementary_tables_s1-3_fdab347.docx]

# Supplementary Table S1. Associations of ethnicity and staff group with Covid-19 sickness absence according to maximum duration of episodes - excluding individuals in whom at least one of sex, age group or ethnicity was imputed because of inconsistencies in original data files

Risk estimates are relative to no Covid-19 sickness absence during study period, and were derived from two logistic regression models (one per outcome), each of which also included trust (200 categories), sex, age group (8 categories), number of episodes of sickness absence in 2019 (4 categories) and exposure category at 9 March 2020 (two categories) – for further detail, see text.

| **Risk factor** | **Covid-19 sickness absence during study period** | | | | | | |
| --- | --- | --- | --- | --- | --- | --- | --- |
|  | **None** | **All episodes ≤14 days** | | | **At least one episode >14 days** | | |
|  | **N** | **N** | **OR** | **(95%CI)** | **N** | **OR** | **(95%CI)** |
|  |  |  |  |  |  |  |  |
| **Ethnicity** |  |  |  |  |  |  |  |
| White | 665,443 | 50,330 | *ref.* | *ref.* | 12,495 | *ref.* | *ref.* |
| Indian | 41,627 | 4,093 | 1.22 | 1.18 - 1.27 | 1,811 | 2.49 | 2.36 - 2.63 |
| Pakistani | 12,091 | 1,090 | 1.10 | 1.03 - 1.17 | 425 | 2.39 | 2.15 - 2.65 |
| Bangladeshi | 4,141 | 348 | 1.17 | 1.04 - 1.31 | 125 | 2.37 | 1.97 - 2.85 |
| South Asian – not further specified | 544 | 50 | 1.04 | 0.76 - 1.42 | 15 | 1.59 | 0.93 - 2.73 |
| Asian – other or unspecified | 31,705 | 5,085 | 1.41 | 1.36 - 1.46 | 2,273 | 2.69 | 2.55 - 2.83 |
| Black – African | 31,470 | 3,144 | 1.04 | 1.00 - 1.08 | 1,422 | 1.82 | 1.71 - 1.94 |
| Black – Caribbean | 13,215 | 1,057 | 0.90 | 0.85 - 0.97 | 451 | 1.39 | 1.26 - 1.54 |
| Black – other or unspecified | 4,499 | 410 | 0.97 | 0.87 - 1.08 | 181 | 1.65 | 1.41 - 1.92 |
| Mixed | 14,164 | 1,442 | 1.09 | 1.02 - 1.15 | 385 | 1.36 | 1.22 - 1.51 |
| Other | 11,342 | 1,346 | 1.24 | 1.17 - 1.32 | 622 | 2.29 | 2.1 - 2.5 |
| Unknown | 31,283 | 2,468 | 1.01 | 0.97 - 1.06 | 783 | 1.27 | 1.18 - 1.37 |
|  |  |  |  |  |  |  |  |
| **Staff group at 9 March 2020** |  |  |  |  |  |  |  |
| Administrative and clerical | 194,135 | 8,781 | *ref.* | *ref.* | 2,340 | *ref.* | *ref.* |
| Additional clinical services | 163,553 | 17,549 | 1.82 | 1.77 - 1.88 | 6,148 | 2.13 | 2.02 - 2.25 |
| Additional professional scientific and technical | 40,050 | 2,407 | 1.38 | 1.31 - 1.44 | 509 | 1.15 | 1.04 - 1.27 |
| Allied health professionals | 65,058 | 6,288 | 1.66 | 1.59 - 1.72 | 1,192 | 1.26 | 1.17 - 1.36 |
| Estates and ancillary | 56,534 | 3,422 | 1.40 | 1.34 - 1.46 | 1,212 | 1.60 | 1.48 - 1.72 |
| Healthcare scientists | 20,625 | 1,229 | 1.19 | 1.12 - 1.27 | 241 | 0.92 | 0.8 - 1.05 |
| Medical and dental | 73,849 | 5,075 | 1.43 | 1.37 - 1.49 | 1,061 | 0.84 | 0.78 - 0.92 |
| Nursing and midwifery registered | 244,276 | 25,809 | 1.81 | 1.76 - 1.86 | 8,232 | 1.83 | 1.74 - 1.94 |
| Other or unknown (including multiple) | 3,444 | 303 | 1.48 | 1.30 - 1.68 | 53 | 1.33 | 1.01 - 1.76 |

# Supplementary Table S2. Associations of ethnicity with short duration Covid-19 sickness absence according to staff group - excluding individuals in whom at least one of sex, age group or ethnicity was imputed because of inconsistencies in original data files

Risk estimates are for Covid-19 sickness absence that was only ever of short duration (≤14 days) relative to no Covid-19 sickness absence, and are derived from separate logistic regression models for each staff group, which also included trust (200 categories), sex, age group (8 categories), number of episodes of sickness absence in 2019 (4 categories) and exposure category at 9 March 2020 (two categories) – for further detail, see text.

| **Ethnic group** | **Staff group** | | | | | | | |
| --- | --- | --- | --- | --- | --- | --- | --- | --- |
|  | **Administrative and clerical** | **Additional clinical services** | **Additional professional scientific and technical** | **Allied health professionals** | **Estates and ancillary** | **Healthcare scientists** | **Medical and dental** | **Nursing and midwifery registered** |
|  | **OR** | **OR** | **OR** | **OR** | **OR** | **OR** | **OR** | **OR** |
|  | **(95%CI)** | **(95%CI)** | **(95%CI)** | **(95%CI)** | **(95%CI)** | **(95%CI)** | **(95%CI)** | **(95%CI)** |
|  |  |  |  |  |  |  |  |  |
| White | *ref* | *ref* | *ref* | *ref* | *ref* | *ref* | *ref* | *ref* |
| South Asian | 1.16 | 1.30 | 1.08 | 0.92 | 1.25 | 1.13 | 0.99 | 1.38 |
|  | 1.05 - 1.27 | 1.21 - 1.41 | 0.92 - 1.26 | 0.8 - 1.06 | 1.05 - 1.49 | 0.92 - 1.37 | 0.92 - 1.07 | 1.3 - 1.45 |
| Asian – other or unspecified | 1.26 | 1.53 | 1.63 | 1.34 | 1.66 | 1.08 | 0.98 | 1.45 |
|  | 1.06 - 1.48 | 1.42 - 1.65 | 1.33 - 1.99 | 1.1 - 1.63 | 1.41 - 1.96 | 0.82 - 1.43 | 0.86 - 1.1 | 1.38 - 1.52 |
| Black | 1.03 | 0.92 | 1.22 | 1.04 | 0.78 | 1.12 | 0.91 | 1.04 |
|  | 0.93 - 1.14 | 0.85 - 0.99 | 0.99 - 1.49 | 0.87 - 1.23 | 0.66 - 0.93 | 0.87 - 1.45 | 0.77 - 1.07 | 0.99 - 1.1 |
| Mixed | 1.13 | 1.06 | 1.20 | 1.21 | 1.08 | 1.16 | 1.01 | 1.05 |
|  | 0.97 - 1.33 | 0.94 - 1.2 | 0.89 - 1.63 | 0.99 - 1.49 | 0.83 - 1.4 | 0.75 - 1.79 | 0.85 - 1.2 | 0.94 - 1.17 |
| Other | 1.23 | 1.34 | 1.13 | 1.13 | 0.75 | 1.25 | 1.06 | 1.29 |
|  | 0.96 - 1.56 | 1.17 - 1.52 | 0.8 - 1.58 | 0.81 - 1.57 | 0.54 - 1.04 | 0.8 - 1.98 | 0.9 - 1.24 | 1.18 - 1.42 |
| Unknown | 1.00 | 1.05 | 0.94 | 0.93 | 0.86 | 1.39 | 0.99 | 1.04 |
|  | 0.88 - 1.14 | 0.96 - 1.15 | 0.72 - 1.22 | 0.79 - 1.11 | 0.71 - 1.03 | 1.04 - 1.87 | 0.87 - 1.13 | 0.96 - 1.13 |

# Supplementary Table S3. Associations of ethnicity with prolonged Covid-19 sickness absence according to staff group - excluding individuals in whom at least one of sex, age group or ethnicity was imputed because of inconsistencies in original data files

Risk estimates are for at least one episode of Covid-19 sickness absence with duration >14 days relative to no Covid-19 sickness absence, and are derived from separate logistic regression models for each staff group, which also included trust (200 categories), sex, age group (8 categories), number of episodes of sickness absence in 2019 (4 categories) and exposure category at 9 March 2020 (two categories) – for further detail, see text.

| **Ethnic group** | **Staff group** | | | | | | | |
| --- | --- | --- | --- | --- | --- | --- | --- | --- |
|  | **Administrative and clerical** | **Additional clinical services** | **Additional professional scientific and technical** | **Allied health professionals** | **Estates and ancillary** | **Healthcare scientists** | **Medical and dental** | **Nursing and midwifery registered** |
|  | **OR** | **OR** | **OR** | **OR** | **OR** | **OR** | **OR** | **OR** |
|  | **(95%CI)** | **(95%CI)** | **(95%CI)** | **(95%CI)** | **(95%CI)** | **(95%CI)** | **(95%CI)** | **(95%CI)** |
|  |  |  |  |  |  |  |  |  |
| White | *Ref* | *ref* | *ref* | *ref* | *ref* | *ref* | *ref* | *ref* |
| South Asian | 1.93 | 2.51 | 2.05 | 1.67 | 2.13 | 3.08 | 1.59 | 3.05 |
|  | 1.64 - 2.26 | 2.26 - 2.79 | 1.53 - 2.73 | 1.28 - 2.16 | 1.67 - 2.72 | 2.16 - 4.4 | 1.37 - 1.85 | 2.82 - 3.29 |
| Asian – other or unspecified | 2.00 | 2.85 | 1.96 | 2.43 | 2.79 | 1.69 | 1.17 | 2.95 |
|  | 1.52 - 2.63 | 2.57 - 3.15 | 1.27 - 3.03 | 1.7 - 3.47 | 2.21 - 3.52 | 0.97 - 2.95 | 0.9 - 1.53 | 2.74 - 3.17 |
| Black | 1.59 | 1.39 | 1.44 | 1.68 | 1.44 | 1.43 | 0.97 | 2.01 |
|  | 1.35 - 1.89 | 1.25 - 1.55 | 0.96 - 2.16 | 1.21 - 2.34 | 1.13 - 1.84 | 0.83 - 2.47 | 0.69 - 1.38 | 1.86 - 2.18 |
| Mixed | 1.44 | 1.22 | 1.23 | 1.10 | 0.98 | 0.30 | 1.23 | 1.57 |
|  | 1.07 - 1.95 | 0.99 - 1.5 | 0.65 - 2.35 | 0.66 - 1.83 | 0.59 - 1.63 | 0.04 - 2.17 | 0.85 - 1.79 | 1.31 - 1.88 |
| Other | 1.87 | 2.55 | 1.55 | 1.32 | 0.73 | 2.98 | 1.54 | 2.62 |
|  | 1.25 - 2.79 | 2.16 - 3.01 | 0.8 - 2.98 | 0.67 - 2.61 | 0.41 - 1.31 | 1.39 - 6.39 | 1.14 - 2.06 | 2.31 - 2.98 |
| Unknown | 1.05 | 1.27 | 1.44 | 0.88 | 1.33 | 1.17 | 1.39 | 1.35 |
|  | 0.82 - 1.34 | 1.1 - 1.46 | 0.9 - 2.31 | 0.61 - 1.26 | 1.02 - 1.74 | 0.56 - 2.46 | 1.07 - 1.81 | 1.18 - 1.54 |
