# Supplementary material for: Ethnic differences in risk of severe Covid-19: To what extent are they driven by exposure?
Source: J Public Health (Oxf). 2021 Sep 21;44(4):787–96. doi: 10.1093/pubmed/fdab347 (PMC8500021; doi:10.1093/pubmed/fdab347)
Supplement: Strobe_Checklist_Paper_2_1_0_fdab347 [file strobe_checklist_paper_2_1_0_fdab347.docx]

STROBE Statement—checklist of items that should be included in reports of observational studies

|  | Item No. | Recommendation | Page  No. | Relevant text from manuscript | |
| --- | --- | --- | --- | --- | --- |
| **Title and abstract** | 1 | (*a*) Indicate the study’s design with a commonly used term in the title or the abstract |  | |  |
|  |  | (*b*) Provide in the abstract an informative and balanced summary of what was done and what was found |  | |  |
| Introduction | | | | |  |
| Background/rationale | 2 | Explain the scientific background and rationale for the investigation being reported |  | | The disproportionate impact of the Covid-19 pandemic on minority ethnic groups in the United Kingdom (UK) is now well established(1), but not fully understood.   During the first wave (24 January 2020 to 11 September 2020), people from all ethnic minority groups (except for women in the Chinese or "White Other" ethnic groups) had higher rates of death involving SARS-CoV-2 than the White British population. The rate was highest for the Black African group (3.7 times greater than for the White British group for males, and 2.6 greater for females), followed by the Bangladeshi (3.0 for males, 1.9 for females), Black Caribbean (2.7 for males, 1.8 for females) and Pakistani (2.2 for males, 2.0 for females) ethnic groups.  These findings could arise from differences in exposure to infection and/or differences in vulnerability to more severe disease when infection occurs. Vulnerability to Covid-19 is related to age, sex, and various comorbidities. One factor that contributes to exposure to SARS-CoV-2 infection is occupation. If minority ethnic groups were employed disproportionately in occupations entailing proximity to other people, particularly people who are more likely to be infected with SARS-CoV-2, then they would be at higher risk of infection.  Exposure to infection will depend also on other factors such as household size and composition, housing density, and non-occupational activities and behaviours.(2) Large record linkage studies such as OpenSAFELY suggest that important differences in mortality by ethnicity persist even after allowance for region, social deprivation, sex, age, and multiple comorbidities.(3) However, it remains possible that there are differences in exposures through work, and to date, few studies have been able to adjust well for occupational differences in exposure. |
| Objectives | 3 | State specific objectives, including any prespecified hypotheses |  | | This study investigates the extent to which infection and with COVID-19 and severe disease among NHS staff differed according to ethnicity during the first wave of the pandemic (March – July 2020). In addition, we will explore whether ethnic disparities in infection and severe disease vary between occupational groups. |
| Methods | | | | |  |
| Study design | 4 | Present key elements of study design early in the paper |  | | As in an earlier report,(4) we analysed pseudonymised data abstracted from the National Health Service (NHS) electronic staff record (ESR) for all personnel who had been continuously employed by NHS trusts in England during 1 January 2019 to 31 July 2020. A detailed account of the sources and initial processing of data is available as an online supplement to that report. |
| Setting | 5 | Describe the setting, locations, and relevant dates, including periods of recruitment, exposure, follow-up, and data collection |  | |  |
| Participants | 6 | (*a*) *Cohort study*—Give the eligibility criteria, and the sources and methods of selection of participants. Describe methods of follow-up  *Case-control study*—Give the eligibility criteria, and the sources and methods of case ascertainment and control selection. Give the rationale for the choice of cases and controls  *Cross-sectional study*—Give the eligibility criteria, and the sources and methods of selection of participants |  | | As in our earlier report [ref], we analysed pseudonymised data abstracted from the National Health Service (NHS) electronic staff record (ESR) for all personnel who had been continuously employed by NHS trusts in England during 1 January 2019 to 31 July 2020. A detailed account of the sources and initial processing of data is available as an online supplement to that report. |
|  |  | (*b*) *Cohort study*—For matched studies, give matching criteria and number of exposed and unexposed  *Case-control study*—For matched studies, give matching criteria and the number of controls per case |  | |  |
| Variables | 7 | Clearly define all outcomes, exposures, predictors, potential confounders, and effect modifiers. Give diagnostic criteria, if applicable |  | | In the analysis for this paper, we focused on two main outcomes – a) Covid-19 sickness absence beginning between 9 March and 16 July 2020, at least one episode of which was prolonged (i.e. with duration >14 days); and b) Covid-19 sickness absence during the same period that was only ever of shorter duration. Covid-19 sickness absence was defined as sickness absence ascribed to any of five diagnostic categories (cough/flu, chest/respiratory, infectious diseases, other, unknown) with Covid-19 recorded as a related reason.  The main explanatory variables of interest were ethnicity and staff group. Ethnicity was classified initially to the 12 categories listed in Table 1, but in some analyses, we aggregated all South Asian ethnic groups and all black ethnic groups to ensure statistically meaningful numbers. Staff group was classed to nine categories (again listed in Table 1), following a scheme that was employed in the ESR, but with students aggregated into a category labelled as “Other or unknown” which also included some individuals who held multiple jobs simultaneously. As in our earlier report [ref], where individuals had changed staff group over the study period, we aimed to classify them according to the job held at 9 March 2020.  In addition, we considered five other explanatory variables – trust (200 categories) sex, age group (8 categories), number of episodes of sickness absence in 2019 (4 categories) and exposure category. The latter was assigned by application of a job-exposure matrix to the occupation (659 possible categories) that the individual held on 9 March 2020. It was assigned to two levels according to whether or not the occupation was judged to involve face-to-face or hands-on care of patients who were more likely to have Covid-19 than the general population. In earlier analyses, such exposure was associated with clearly elevated risk of Covid-19 sickness absence [ref]. The other variables were classified as in our previous report [ref]. |
| Data sources/ measurement | 8* | For each variable of interest, give sources of data and details of methods of assessment (measurement). Describe comparability of assessment methods if there is more than one group |  | | National Health Service (NHS) electronic staff record (ESR) for all personnel who had been continuously employed by NHS trusts in England during 1 January 2019 to 31 July 2020 |
| Bias | 9 | Describe any efforts to address potential sources of bias |  | |  |
| Study size | 10 | Explain how the study size was arrived at |  | |  |

Continued on next page

| Quantitative variables | 11 | Explain how quantitative variables were handled in the analyses. If applicable, describe which groupings were chosen and why |  |  |
| --- | --- | --- | --- | --- |
| Statistical methods | 12 | (*a*) Describe all statistical methods, including those used to control for confounding |  | Statistical analysis was carried out with R statistical software. We used logistic regression to estimate odds ratios (ORs) with 95% confidence intervals (CIs) for the two outcomes in relation to combinations of ethnicity and staff group with adjustment for other explanatory variables. |
|  |  | (*b*) Describe any methods used to examine subgroups and interactions |  |  |
|  |  | (*c*) Explain how missing data were addressed |  |  |
|  |  | (*d*) *Cohort study*—If applicable, explain how loss to follow-up was addressed  *Case-control study*—If applicable, explain how matching of cases and controls was addressed  *Cross-sectional study*—If applicable, describe analytical methods taking account of sampling strategy |  |  |
|  |  | (*e*) Describe any sensitivity analyses |  |  |
| Results | | | | |
| Participants | 13* | (a) Report numbers of individuals at each stage of study—eg numbers potentially eligible, examined for eligibility, confirmed eligible, included in the study, completing follow-up, and analysed |  | After exclusion of 3,811 employees who were absent from work continuously between 9 March and 31 July 2020 (mainly because of maternity or study leave), analysis was based on 959,356 individuals (77% female) from 200 trusts. |
|  |  | (b) Give reasons for non-participation at each stage |  |  |
|  |  | (c) Consider use of a flow diagram |  |  |
| Descriptive data | 14* | (a) Give characteristics of study participants (eg demographic, clinical, social) and information on exposures and potential confounders |  | Most (89%) were aged between 25 and 60 years. Detailed information on the numbers of individuals by age band and by frequency of sickness absence during 2019 has been reported elsewhere [ref]. From application of the job-exposure matrix, 383,097 (39.9%) subjects held jobs at 9 March 2020, which were classed as providing hands-on or face-to face care for patients who could be expected to have a higher prevalence of Covid-19 than the general population. Table 1 shows the distribution of the study sample according staff group at 9 March 2020 and ethnic group. Among staff of Asian origin, the proportion employed as doctors or dentists was some five times higher than in white workers. Relatively high proportions of the black ethnic groups, and especially black African staff, were registered nurses or midwives. |
|  |  | (b) Indicate number of participants with missing data for each variable of interest |  |  |
|  |  | (c) *Cohort study*—Summarise follow-up time (eg, average and total amount) |  |  |
| Outcome data | 15* | *Cohort study*—Report numbers of outcome events or summary measures over time |  |  |
|  |  | *Case-control study—*Report numbers in each exposure category, or summary measures of exposure |  |  |
|  |  | *Cross-sectional study—*Report numbers of outcome events or summary measures |  | In total, 20,988 individuals (2.2%) had at least one episode of Covid-19 sickness absence that started between 9 March and 16 July 2020, and continued for >14 days (prolonged Covid-19 sickness absence). In addition, a further 70,863 (7.4%) had episodes of Covid-19 sickness absence during that period, all of which were of shorter duration. |
| Main results | 16 | (*a*) Give unadjusted estimates and, if applicable, confounder-adjusted estimates and their precision (eg, 95% confidence interval). Make clear which confounders were adjusted for and why they were included |  | In comparison with white ethnicity, risk of short-duration Covid-19 sickness absence was modestly elevated in all other ethnic groups except black Caribbean (ORs up to 1.41. Moreover, non-white ethnic groups were also at higher risk of prolonged Covid-19 sickness absence, but to a greater extent. In particular, ORs relative to white ethnicity were more than doubled for those of South Asian origin, while that for black Africans was 1.82. Within those of South Asian ethnicity, there was little difference in risk between those with origins in India, Pakistan and Bangladesh.  Risk estimates are relative to no Covid-19 sickness absence during study period, and were derived from two logistic regression models (one per outcome), each of which also included trust (200 categories), sex, age group (8 categories), number of episodes of sickness absence in 2019 (4 categories) and exposure category at 9 March 2020 (two categories) – for further detail, see text. |
|  |  | (*b*) Report category boundaries when continuous variables were categorized |  | Table 2 shows associations of Covid-19 sickness absence with ethnicity and staff group, according to whether absence was only ever of short duration (≤14 days), or at least one episode was prolonged. |
|  |  | (*c*) If relevant, consider translating estimates of relative risk into absolute risk for a meaningful time period |  |  |

Continued on next page

| Other analyses | 17 | Report other analyses done—eg analyses of subgroups and interactions, and sensitivity analyses |  | In sensitivity analyses, we repeated the calculations for Tables 2 to 4, after exclusion of 6,854 individuals for whom one or more of age, sex or ethnicity was imputed because of inconsistencies in the raw data. The results, which are presented in Supplementary Tables S1 to S3, were virtually unchanged. |
| --- | --- | --- | --- | --- |
| Discussion | | | | |
| Key results | 18 | Summarise key results with reference to study objectives |  | Our analysis confirms that during the first wave of Covid-19 in England there were differences between ethnic groups in risk of short and longer duration Covid-19 sickness absence amongst NHS staff. Once staff group, age, sex, prior sickness absence, trust and occupational exposure category were accounted for, the risk of short duration Covid-19 was similar for Black people compared with White, and only marginally elevated for people of South Asian origin. In contrast staff from Black and other ethnic minority groups were at a higher risk of prolonged Covid-19 sickness absence compared to White NHS employees suggesting important ethnic differences in vulnerability, whether because of comorbidities or for other reasons. |
| Limitations | 19 | Discuss limitations of the study, taking into account sources of potential bias or imprecision. Discuss both direction and magnitude of any potential bias |  | Ethnicity was coded in the electronic staff record with varying degrees of specificity and not always consistently. Exposure category was defined based on employment at 9th March 2020 and did not capture redeployment to different clinical settings during the pandemic. We acknowledge that we were not able to account for use of personal protective equipment which may have biased our analysis if it differed by ethnicity within job groups. A British Medical Association snapshot survey taken early in the first wave of the pandemic suggested that a higher proportion (68%) of doctors from minority ethnic groups felt pressured to work with inadequate personal protective equipment where aerosol-generating procedures were being carried out, than those who identified as White (33%).(11) A further limitation is that sickness absence is an imperfect marker for the occurrence of Covid-19, and it is possible both that true cases were missed (due to asymptomatic illness) and that other respiratory illnesses were sometimes incorrectly attributed to coronavirus. However, a previous analysis on the same dataset showed that Covid-19 sickness absence correlated with seropositivity for SARS-Cov-2.(4) |
| Interpretation | 20 | Give a cautious overall interpretation of results considering objectives, limitations, multiplicity of analyses, results from similar studies, and other relevant evidence |  | This large study is the first to examine the associations of ethnicity with Covid-19 sickness absence in UK healthcare workers while accounting for occupational group and potential for exposure to infected patients. The sample size of almost a million individuals gave the investigation high statistical power and allowed us to investigate ethnic groups in detail (for example, separating workers of Indian and Pakistani origin). Occupational groups were analysed separately, and an attempt was made to adjust for occupational exposure by using a bespoke job-exposure matrix. The effect of geographical differences in exposure to infection was accounted for by adjustment for hospital trust.  We explored the risk of less serious Covid-19 among NHS staff (as measured by short-duration sickness absence attributed to Covid-19), which is less likely to be influenced by differences in vulnerability. By adjusting for the potential occupational exposure to infected patients (assessed by the job-exposure matrix), as well as trust (a more specific geographical marker than region), sex and age, we have shown that any differences in risk of mild Covid-19 by ethnicity were small.  The residual variation may reflect differences in exposure that were not adequately captured by staff group and exposure category.  In contrast, the difference in risk of prolonged Covid-19 amongst Black and ethnic minority groups compared to White was more exaggerated than for short duration Covid-19 sickness absence. Within each staff group, the risk of prolonged Covid-19 sickness absence was highest in the South Asian and/or the other/unspecified Asian ethnic groups, and often the odds were twice those of White people. Our findings that ethnic minority groups are at higher risk of severe Covid-19 is supported by several other studies.  In our study, ethnic disparities in short duration Covid-19 sickness absence were not observed amongst those employed as healthcare scientists or doctors and dentists, in contrast to those employed in other roles within the NHS. It may be that non-occupational risk factors for infection differ less by ethnicity within these groups than in other job groups. Within healthcare scientists, doctors and dentists, ethnic differences were apparent, however, for longer duration Covid-19 sickness absence, again suggesting differences in vulnerability to severe illness when infection occurs |
| Generalisability | 21 | Discuss the generalisability (external validity) of the study results |  |  |
| Other information | |  | | |
| Funding | 22 | Give the source of funding and the role of the funders for the present study and, if applicable, for the original study on which the present article is based |  | This work was supported by Colt Foundation UK |

*Give information separately for cases and controls in case-control studies and, if applicable, for exposed and unexposed groups in cohort and cross-sectional studies.

**Note:** An Explanation and Elaboration article discusses each checklist item and gives methodological background and published examples of transparent reporting. The STROBE checklist is best used in conjunction with this article (freely available on the Web sites of PLoS Medicine at http://www.plosmedicine.org/, Annals of Internal Medicine at http://www.annals.org/, and Epidemiology at http://www.epidem.com/). Information on the STROBE Initiative is available at www.strobe-statement.org.
